# lpxC and yafS are the Most Suitable Internal Controls to Normalize Real Time RT-qPCR Expression in the Phytopathogenic Bacteria *Dickeya dadantii*

**DOI:** 10.1371/journal.pone.0020269

**Published:** 2011-05-26

**Authors:** Florence Hommais, Ouafa Zghidi-Abouzid, Christine Oger-Desfeux, Emilie Pineau-Chapelle, Frederique Van Gijsegem, William Nasser, Sylvie Reverchon

**Affiliations:** 1 Unité Microbiologie, Adaptation, Pathogénie CNRS-INSA-UCBL UMR 5240, Université Lyon 1, Villeurbanne, France, Université de Lyon, France; INSA-Lyon, Villeurbanne, France; 2 Pôle Rhône-Alpes de Bioinformatique, IFR41, Université Lyon 1, Batiment G. Mendel, Villeurbanne, France, Université de Lyon, France; 3 Laboratoire Interactions Plantes Pathogènes, UMR 217 INRA/AgroParisTech/UPMC P6, Paris, France; University of Wisconsin-Milwaukee, United States of America

## Abstract

**Background:**

Quantitative RT-PCR is the method of choice for studying, with both sensitivity and accuracy, the expression of genes. A reliable normalization of the data, using several reference genes, is critical for an accurate quantification of gene expression. Here, we propose a set of reference genes, of the phytopathogenic bacteria *Dickeya dadantii* and *Pectobacterium atrosepticum*, which are stable in a wide range of growth conditions.

**Results:**

We extracted, from a *D. dadantii* micro-array transcript profile dataset comprising thirty-two different growth conditions, an initial set of 49 expressed genes with very low variation in gene expression. Out of these, we retained 10 genes representing different functional categories, different levels of expression (low, medium, and high) and with no systematic variation in expression correlating with growth conditions. We measured the expression of these reference gene candidates using quantitative RT-PCR in 50 different experimental conditions, mimicking the environment encountered by the bacteria in their host and directly during the infection process *in planta*. The two most stable genes (ABF-0017965 (*lpxC*) and ABF-0020529 (*yafS*) were successfully used for normalization of RT-qPCR data. Finally, we demonstrated that the ortholog of *lpxC* and *yafS* in *Pectobacterium atrosepticum* also showed stable expression in diverse growth conditions.

**Conclusions:**

We have identified at least two genes, *lpxC* (ABF-0017965) and *yafS* (ABF-0020509), whose expressions are stable in a wide range of growth conditions and during infection. Thus, these genes are considered suitable for use as reference genes for the normalization of real-time RT-qPCR data of the two main pectinolytic phytopathogenic bacteria *D. dadantii* and *P. atrosepticum* and, probably, of other *Enterobacteriaceae*. Moreover, we defined general criteria to select good reference genes in bacteria.

## Introduction

Gene expression analysis is becoming more important in many biological fields, such as applied functional genomic research and the study of infection processes. Consequently the quantification of gene expression has to be accurate. Due to its high sequence-specificity and its tremendous sensibility, quantitative real-time RT-PCR (RT-qPCR) has become the method of choice for quantifying the expression of selected genes in an increasing number of biological samples. However, noisy technical variations, such as RNA extraction efficiency, RNA quality or cDNA synthesis efficiency, in different biological samples could interfere with the final expression measurements. Consequently, data generated with RT-qPCR should be normalized to compensate for these variations. The most frequently used method to minimize such variations is relative normalization, where the expression of a target gene is quantified with respect to stably expressed internal reference genes. Indeed, recent studies demonstrated that only the use of several reference genes results in accurate normalization [1]. Genes used for normalization should be expressed stably in the conditions of interest. Housekeeping genes have been commonly used as reference genes. Indeed, these genes are described as being essential and ubiquitous. Unfortunately, housekeeping genes have recently been demonstrated to be highly variable under several experimental conditions [2]. The use of such inappropriate reference genes in the relative quantification of gene expression could result in biased expression profiles. Therefore, finding stable reference genes is becoming an essential prerequisite for a reliable measurement of gene expression.

*Dickeya dadantii* (ex *Erwinia chrysanthemi*, [3]) is described as a macerogenic Gram-negative plant pathogen that causes disease in a wide range of plant species, including many crops of economic importance such as vegetables, ornamentals and also the model plant *Arabidopsis thaliana* [4], [5]. This bacterium is associated with the production of pectinases that cleave the pectic component of plant cell walls. The infection process of *D. dadantii* begins with a colonization phase where the bacteria reside within the intracellular spaces, without causing symptoms, until favorable conditions for infection occur. The symptomatic phase that follows consists of the production of plant cell wall degrading enzymes and a massive bacterial multiplication [6], [7]. The genome sequence of *D. dadantii* model 3937 strain is available in genbank (accession number CP002038). This has provided valuable tools for gene expression analysis during plant infection, both at genome-wide levels (with micro-arrays) or at single-gene levels (with quantitative real-time RT-PCR) [8], [9], [10].

Using micro-array transcript profiling, we have tested the expression stability of the 4753 genes of the *D. dadantii* genome under thirty-two different growth conditions. We present the selection of ten potential candidate reference genes, out of an initial pool of 49 genes, with expression above a minimal mean level and very low variation. Quantitative real-time RT-PCR has been used to validate their expression stability in 48 experimental conditions and during the infection process in *Arabidopsis thaliana* plants. The use of these genes for the normalization of RT-qPCR data was analyzed. Subsequently, the stability of 5 orthologous genes in *Pectobacterium atrosepticum* was assessed.

## Results and Discussion

### Selection of reference gene candidates

**Figure 1:**
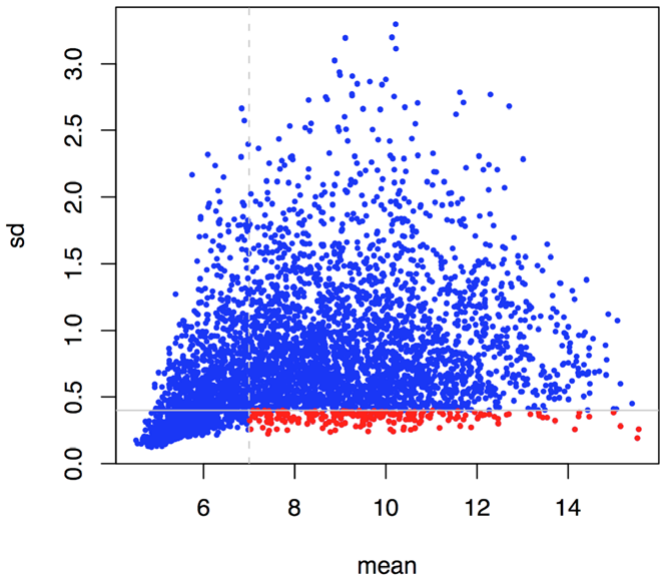
Mean versus standard deviation of gene expression levels in *D. dadantii* measured from micro-arrays. Points in red correspond to genes with a mean expression level above 7 and a standard deviation below 0.4.

In order to select valid candidate reference genes, we used CDS pangenomic micro-arrays to study the *D. dadantii* 3937 transcriptome. Thirty-two different growth conditions were chosen to mimic the environment encountered by the bacteria in its host, as described in the Material and Methods section. These included bacterial growth conditions in the presence of leaf extracts and/or Polygalacturonate (PGA), and stress conditions such as acid, oxidative or osmotic shocks. In order to select the most stably expressed genes, we first retained the genes with a mean expression level above 7 (in log2, this value was chosen arbitrarily as it was slightly superior to the mode for non-expressed genes). Then, in order to select genes with the least variable expression, standard deviations and differences between the highest and the lowest expression (max-min) were calculated for each gene. 209 genes were selected with a mean expression level above 7 and standard deviation below 0.4 (Figure 1, in red). This data demonstrates that such low variability genes span a wide range of expression level, from 7 to almost 16. Setting a threshold of max-min expression of 1.5 (in log2), we selected an initial set of 49 genes from among the previous 209 candidate genes (Table 1). Eighteen of them had a mean expression level between 7 and 9, twenty-six between 9 and 11, and five between 11 and 14.

**Table 1:** Top 49 genes selected for their expression stabilities measured from the micro-array dataset.

Mean of expression level	Accession number	Gene name	Annotation
Low expression level (log>7)	14832	*ygiF*	Predicted adenylate cyclase
	15002	*hemY*	Predicted protoheme IX synthesis protein
	16189	<NA>	Probable formyl-coenzyme A transferase
	16292	*mobA*	Molybdopterin-guanine dinucleotide biosynthesis protein A
	16340	<NA>	Transcription regulator
	16480	<NA>	Bll6904 protein
	17101	<NA>	Phenolic acid decarboxylase
	17576	*yajL*	Conserved protein
	17758	*ymfB*	Thiamin pyrophosphate (TPP) hydrolase
	17970	*yacG*	Zinc-binding protein
	18245	<NA>	Putative cytoplasmic protein
	**18436**	**<NA>**	**Hypothetical protein**
	**18449**	**<NA>**	**Hypothetical protein**
	18939	<NA>	Transcriptional repressor of the fructose operon, DeoR family
	19898	*nlp*	Putative regulator for maltose metabolism
	19985	<NA>	Carbonic anhydrase
	**20529**	***yafS***	**Predicted S-adenosyl-L-methionine-dependent methyltransferase**
	46549	*yjiE*	Putative transcriptional regulator LYSR-type
Middle expression level (log>9)	14861	*relA*	GTP pyrophosphokinase/Guanosine-3′,5′-bis(diphosphate) 3′-pyrophosphohydrolase
	15003	*hemX*	Predicted uroporphyrinogen III methylase
	15008	*cyaY*	Frataxin homolog CyaY, facilitates iron supply for heme A synthesis or Fe-S cluster assembly
	**15073**	***glpR***	**DNA-binding transcriptional repressor**
	**15447**	***yadH***	**Predicted transporter subunit: membrane component of ABC superfamily**
	**16418**	***ddlA***	**D-alanine–D-alanine ligase**
	16646	<NA>	Hypothetical protein
	**16748**	***hemF***	**Coproporphyrinogen III oxidase**
	16791	*yjeH*	Predicted transporter
	16977	*ybfF*	Esterase
	17624	<NA>	Type I restriction-modification system methyltransferase subunit
	17766	*ycfD*	Hypothetical protein
	18061	*ygfZ*	Predicted folate-dependent regulatory protein
	18496	*yggS*	Predicted enzyme
	18606	*nadR*	NadR transcriptional repressor/ribosylnicotinamide kinase/NMN adenylyltransferase
	18965	*minE*	Cell division topological specificity factor
	19439	*yicC*	Conserved protein
	20070	*ycbB*	Predicted carboxypeptidase
	20153	<NA>	Hypothetical protein
	20393	*yiiQ*	Conserved protein
	**20403**	***rraA***	**Ribonuclease E (RNase E) inhibitor protein**
	20510	*pgsA*	CDP-diacylglycerol–glycerol-3-phosphate 3-phosphatidyltransferase
	20531	*dnaQ*	DNA polymerase III epsilon subunit
	20751	*manA*	Mannose-6-phosphate isomerase
	20824	*damX*	Membrane protein
	47165	*gloA*	Glyoxalase I, Ni-dependent
High expression level (log>11)	**15677**	***yhbN***	**Periplasmic component of an ABC-type LPS transporter complex**
	**16832**	***queF***	**NADPH dependent preQ0 reductase**
	**17965**	***lpxC***	**UDP-3-O-acyl N-acetylglucosamine deacetylase**
	19603	*focA*	fFrmate transporter
	20671	*yadG*	Predicted transporter subunit: ATP-binding component of ABC superfamily

In bold the 10 genes selected for further investigation.

**Figure 2:**
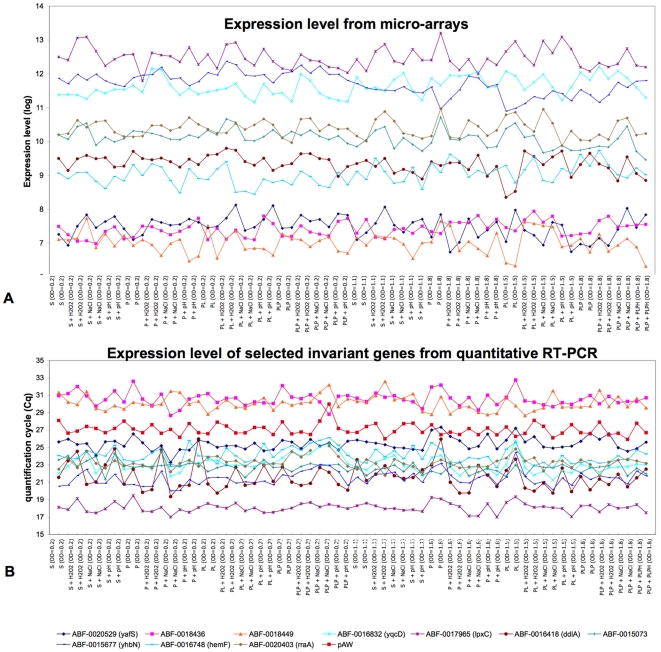
Expression profiles of the 10 genes selected for the stability of their expression level and the synthetic RNA pAW. Gene expression profiles are measured from micro-arrays (A) and from quantitative real time RT-PCR (B). The first 32 conditions are those measured in exponential phase and the last 32 conditions are those measured in stationary phase. The order of conditions is M63 supplemented with saccharose (S), M63 supplemented with saccharose and Saintpaulia leaf extract (PL), M63 supplemented with saccharose and PGA (P), and M63 supplemented with saccharose, leaves and PGA (PLP). The order of stress is: no stress, oxidative stress, acid stress, and osmotic stress.

Next, we required that the selected genes satisfied the following criteria: (i) they are associated, as far as possible, with different functional categories to minimize the risk of choosing co-regulated genes; (ii) they belong to different transcription units; (iii) they show no sign of correlation between the expression profiles and growth conditions (supplementary data). Among the 49 genes, 22 encode metabolic enzymes, 9 genes encode membrane proteins involved in membrane traffic and 6 are predicted to encode transcriptional regulators, the others encoding hypothetical proteins or proteins with unknown function. No functional category is overrepresented, with the exception of three genes which encode proteins involved in heme biosynthesis: *hemF* and *hemXY*. One of the most stable genes is *lpxC* (ABF-0017965). This gene encodes for a UDP-3-O-acyl N-acetylglucosamine deacetylase that catalyzes the second step of the lipid A-precursor biosynthetic pathway [11]. Seven enzymes are involved in this pathway and most of the encoding genes are significantly down-regulated in oxidative stress when grown at the exponential phase, suggesting that the expression of *lpxC* (ABF-0017965) is stable whereas that of the other genes from the same pathway is under regulation. This means that enzymes from the same metabolic pathway may be differentially regulated. Among the 49 selected genes, only *hemY*, *hemX* and *yadH*, *yadG* are organized in operons. *hemXY* are transcribed with two other genes, *hemC* and *hemD*. Remarkably, expression of *hemCD* is induced by osmotic stress (1.5 log), suggesting a difference in the regulation mechanisms between *hemXY* and *hemCD*. This is in accordance with a previous study, using *E. coli*, that demonstrates the presence of two transcription initiation sites in this operon [12]. The expression of the two other genes organized in operons, *yadH* and *yadG*, is very similar (supplementary data) and seems to be correlated to growth phases and stresses. These genes were discarded for the rest of the study. We examined the expression profiles of the 47 genes (supplementary data) and checked for systematic variation and correlations between expression and growth conditions. In spite of the weak variation in the expression levels of the selected genes, some correlations were found between the expression levels of several genes and the growth conditions tested. For example, the expression level of ABF-0020153 is weakly, but significantly, induced by oxidative or osmotic stresses (supplementary data). Such genes were discarded for the remainder of the study. Eventually we retained, for further investigation, ten genes satisfying the criteria above. Three of these genes display a low expression level (mean of log expression level below 9): ABF-0018436, ABF-0018449 and ABF-20529 (*yafS*). Three others have high expression levels (mean of log expression level above 11): ABF-0016832 (*yqcD*), ABF-0017965 (*lpxC*) and ABF-0015677 (*yhbN*). Finally, four genes have an intermediate expression level (mean of log expression level between 9 and 11): ABF-0020403 (*rraA*), ABF-0015073, ABF-0016418 (*ddlA*) and ABF-0018748. Their expression level profiles, measured from micro-arrays, are presented in Figure 2A.

### Expression analysis of reference genes by RT-qPCR

**Figure 3:**
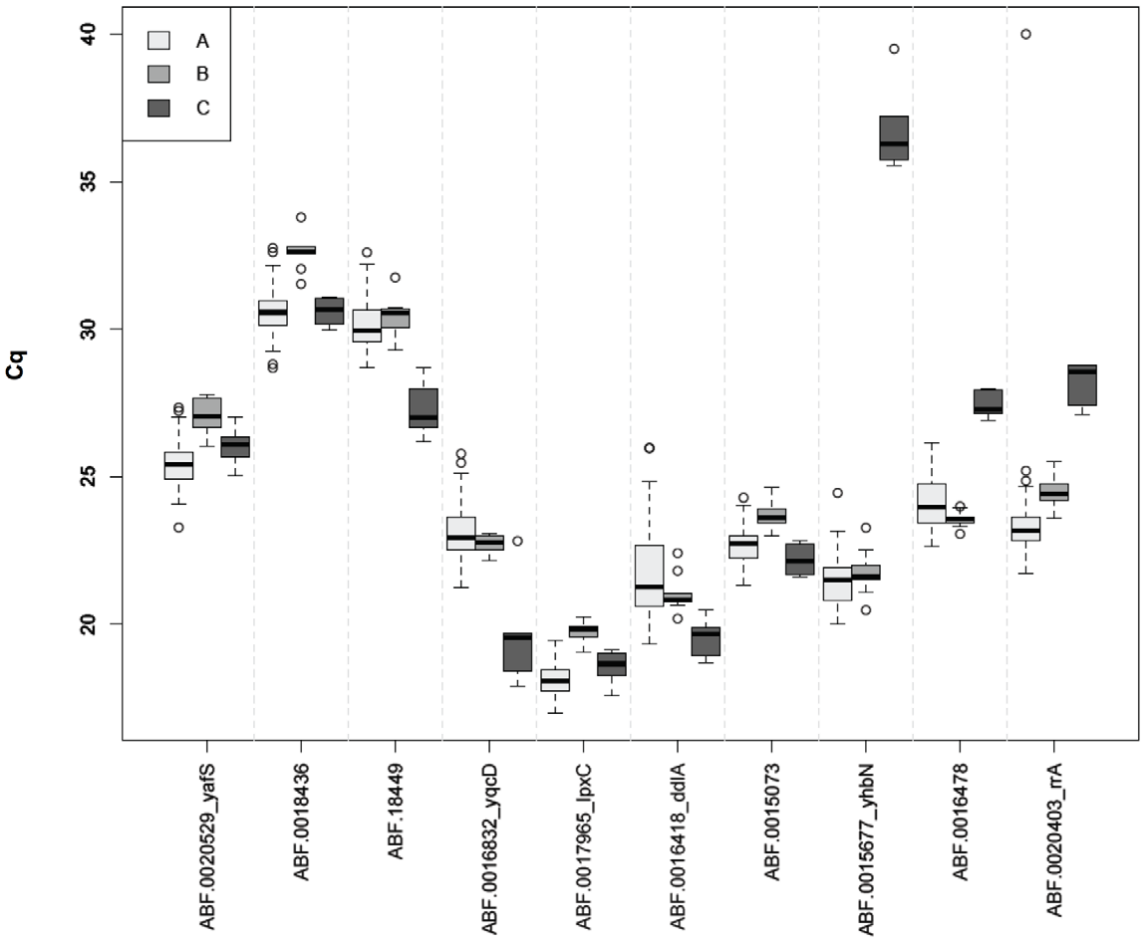
RT-qPCR results of the 10 candidate reference genes. Results are represented by boxplots of the Cq values, measured from quantitative real time RT-qPCR, for the first 32 growth conditions in duplicates (white, A), the 10 growth conditions with different carbon sources (grey, B), and the 6 growth conditions modulating the supercoiling state of DNA (black, C).

None of these 10 selected genes had been previously used to normalize expression data. To validate their use as reference genes, transcription profiling, using real-time RT-qPCR assays, was performed and the invariability expression of these ten genes was evaluated in the 32 experimental conditions previously tested in micro-arrays (with two biological replicates). The results are presented in Figure 2B. Since an equal quantity of total RNA was used in each reaction, we directly compared transcript abundances using quantitative Cycle results, previously known as the threshold cycle Ct, crossing point CP, or take-off point TOP [13]. For the ten genes, the Cq values ranged from 17.99 to 32.75. In accordance with the micro-arrays results, the lowest expressed genes are ABF-0018436 and ABF-0018449 with a mean Cq value of 30.56 and 30.09, respectively. The highest expressed gene is ABF-0017965 (*lpxC*) with a mean Cq of 18.10. Most of the genes showed low variation of their Cq values but this is not the case with ABF-0016418, the Cq values of which range from 19.36 to 25.98 (Figure 3). Hence this does not seem to be a good reference gene for the normalization of expression data in these growth conditions. In order to evaluate the stability of each candidate, and to discriminate between technical and biological variability, a total of 2×10^5^ copies of GeneAmplimer pAW 109 RNA were added to the reverse transcriptional reaction mixture and used as a control of experimental efficiency (Applied Biosystems) [8], [10], [14]. Standard deviation of pAW expression level in the quantitative Cycle (Cq) was evaluated from the 64 reactions and a standard deviation of Cq (ΔCq) equal to 0.4 was obtained, showing weak technical variability in our samples. Except for ABF-0016418, with a ΔCq of 1.22, the ΔCq values of the candidate reference genes were comparable to those of pAW which means that the Cq variation observed could be attributed to technical variability.

To statistically identify the most suitable candidate genes, we analyzed our data using geNorm and Bestkeeper algorithms [1], [15], [16]. The geNorm principle for selecting candidate reference genes is that the expression ratio of two ideal reference genes should be constant across the samples used. For each candidate reference gene, its pairwise variation is measured with all the other candidate genes. Genes are ranked according to the gene stability value M, which is the average pairwise variation of that gene with all the other candidate reference genes. Genes are arranged from the least variable to the most variable, according to geNorm results: ABF-0017965 (*lpxC*), ABF-0015073, ABF-0020529 (*yafS*), ABF-0018436, ABF-0015677 (*yhbN*), ABF-0018449, ABF-0016832 (*yqcD*), ABF-0016748, and ABF-0020403 (*rraA*) (Table S1). Results were also analyzed using the Bestkeeper algorithm (Table S2). In spite of slight differences between the results obtained, both approaches agreed on the more stable reference genes. Taking this into account, the nine reference genes: ABF-0017965 (*lpxC*), ABF-0015073, ABF-0020529 (*yafS*), ABF-0018436, ABF-0015677 (*yhbN*), ABF-0018449, ABF-0016832 (*yqcD*), ABF-0016748, and ABF-0020403 (*rraA*), are suitable for the normalization of expression data in this set of experimental conditions.

### Stability measurement of reference genes in cultures *and in planta*

**Figure 4:**
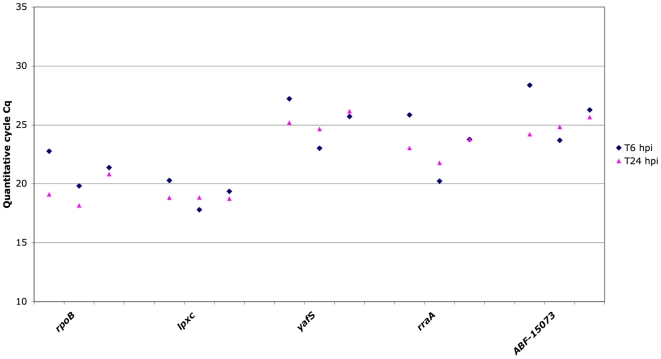
Expression profiles of the selected invariant genes during *A. thaliana* infection by *D. dadantii*. Gene expression was measured by RT-qPCR on bacterial RNA samples recovered at two distinct time points during the infection process: T 6 hpi samples correspond to the population recovered 6 hpi (hours post-infection), during the early asymptomatic phase (in blue), and T 24 hpi corresponds to the whole bacterial population recovered 24 hpi from macerated plant tissues (in red). Values from three independent experiments are presented. Five genes were analyzed: *yafS, lpxC*, ABF-0020403, ABF-0015073 and *rpoB*, classically used for normalization of *D. dadantii* gene expression during the infection process.

It is now acknowledged that no gene has a constant expression level regardless of the experimental conditions tested. Every gene is regulated and the expression level may vary slightly. In order to expand the use of the nine reference genes identified here, we determined their expression in new experimental conditions, as follows: exponential and stationary phases in rich medium, as used routinely in the laboratory (LB with or without PGA, Poly Galacturonic Acid), and minimal medium supplemented with different carbon sources normally encountered in host plants or in the environment, e.g. xylose, galactose or rhamnose. In addition to these conditions, we also considered the DNA supercoiling state since recent data shows that this parameter modulates virulence gene expression [17]. Transcript accumulation of the retained genes was thus determined in the presence of Coumermycin, a compound which relaxes DNA, in both exponential and stationary phases of growth. We finally extended these studies to the *in planta* conditions by monitoring the expression levels of the candidate genes during infection of *Arabidopsis thaliana* by *D. dadantii*. For this experiment, *D. dadantii* cells were purified from infected plantlets at two very different time points: 6 hours post-infection (6 hpi), corresponding to the early stage of infection (before a significant detection of virulence gene transcripts) and 24 hours post-infection (24 hpi), corresponding to the maceration stage (after the induction of virulence gene transcript production) [7]. Of the nine initially retained genes, only four showed no sequence similarity in the genome of *A. thalinia* (ABF-0017965 (*lpxC*), ABF-0020529 (*yafS*), ABF-0020403 and ABF-0015073). Hence we only retained these four genes for the *in planta* studies. The *rpoB* gene was also analyzed as it is classically used as a reference gene for *in planta* normalization [7]. Thus, eighteen additional growth conditions were tested. In all these conditions, Cq values ranged from 17.59 to 39.50 (Figures 3 and 4). Expression stability was evaluated for each gene with BestKeeper and geNorm algorithms (Tables S1 and S2). As expression in culture and *in planta* cannot be equalized and directly compared [18], analyses were made separately. In accordance with the BestKeeper analysis, the classification obtained by geNorm showed that the most stable genes in these new laboratory growth conditions are: ABF-0017965 (*lpxC*), ABF-0016748 (*hemF*), ABF-20403 (*rraA*), ABF-0015073 and ABF-0020529 (*yafS*) (Tables S1 and S2), also indicating that these genes are suitable for use as reference genes.

Since contamination with plant RNA is negligible and an equal quantity of total RNA was used in each reaction, we directly compared transcript abundances using the Cq results. Standard deviations ranged from 0.56 (*lpxC*) to 1.39 (ABF-0020403, *rraA*). These expression data were statistically analyzed. The combination of geNorm and Bestkeeper analyses indicated that ABF-0017965 (*lpxC*) and ABF-20529 (*yafS*) are the most stable genes during the infection process and, thus, better reference genes when compared to ABF-0020403, *rraA*, ABF-0020403 or *rpoB*. Both genes could be used as reference genes for the normalization of expression analysis during the infection process. The study of gene expression during pathogen-host interactions is really complex, as RNA can be masked by the higher concentration of host RNA and usually the total pathogen RNA concentration cannot be reliably measured. Here we have demonstrated that the two candidate genes, *lpxC* (ABF-0017965) and *yafS* (ABF-0020529), are stably expressed during the infection and could be used as a marker of the bacterial population size.

### Use of selected reference genes to normalize expression of differentially expressed genes in stress conditions

**Figure 5:**
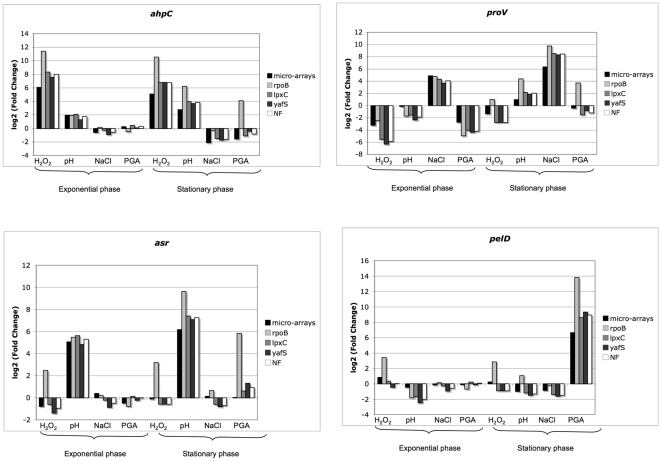
Relative expression levels of selected target genes. The relative expression levels of the four genes, *ahpC*, *proV*, *asr* and *pelD*, were measured and calculated following different normalization strategies: with *rpoB* in pale gray, with *lpxC* alone in gray, with *yafS* alone in dark gray and with the geometric mean of *lpxC* and *yafS* in white. Growth conditions were chosen to induce at least one of the four marker genes.

Previously, several reference genes have been proposed for normalization, such as *rpoAB*, *ffh* or *rsmA* [8], [10], [19], [20]. The reference gene dataset identified in this study was used in assays to further explore the expression of differentially expressed genes. The results of the normalizations obtained were compared with those obtained when *rpoB* was used for normalization. The relative expression level of selected target genes was analyzed using the following three different normalization strategies: i) the frequently used reference gene *rpoB* ii) the two more stable reference genes selected by geNorm and Bestkeeper, *lpxC* and *yafS*, were used individually or, iii) the geometric mean of the top ranking genes selected by geNorm and Bestkeeper methods, *lpxC* and *yafS*. For this purpose, four target genes were selected that are known to be differentially expressed in cultures: *ahpC* is described as being induced in oxidative stress, *proV* is induced in osmotic stress, *asr* is induced in acid stress, and *pelD* is induced in the presence of PGA in the stationary phase. The expression of the genes of interest was evaluated according to the different normalization processes using REST software [21] (Figure 5). As expected, a significant increase in *ahpC*, *asr* and *proV* expressions were observed in oxidative, acid and osmotic stress conditions, respectively. In all cases, normalization by *rpoB*, *lpxC yafS* or the geometric mean of *lpxC* and *yafS* gave results similar to those obtained with the micro-arrays data. However, the induction of *ahpC* is overestimated when normalized with *rpoB* compared to the other normalization strategies. Similar overestimations were also observed for *asr* and *pelD* when normalization was performed with *rpoB* in oxidative stress and in the presence of PGA at the stationary growth phase. Consistently, the analysis of *rpoB* expression profiles by micro-arrays demonstrated that this gene is always repressed by oxidative stress, as well as by acid shock and by the presence of PGA during the stationary phase of growth (Figure S1). This could explain the differences observed in the normalization with *rpoB*, compared to the other normalization strategies, and suggests that *rpoB* is not suitable for normalization of gene expression levels, at least in the growth conditions used here. Finally, these results revealed how an estimation of bacterial gene expression levels can be affected by the choice of the reference genes in quantitative real-time RT-PCR analysis.

### Stability of orthologous genes in *Pectobacterium atrosepticum*

**Figure 6:**
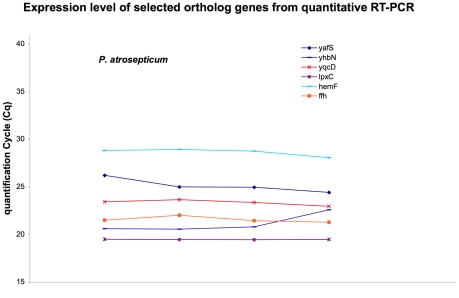
Expression profiles of genes measured on RNA extracted from Pectobacterium atrosepticum cells grown in different conditions. Four different growth conditions were tested, with cells grown in minimal medium and in rich medium. Six genes were analyzed: *yafS*, *lpxC*, *yhbN*, *yqcD*, *hemF* and *ffh*.

Recently, an evaluation of reference genes for normalization was published concerning *P. atrosepticum*. Two genes, *ffh* and *recA*, were proposed as being the most stable genes in the conditions tested (exponential and stationary growth phases in three different growth media -with or without pectin- at 15°C or 27°C) [18]. Unfortunately, the expression of *recA* was shown to be significantly increased by oxidative stress and that of *ffh* by osmotic stress in the *D. dadantii* micro-array dataset (supplementary data). To extend the usefulness of our proposed reference genes, we searched for their orthologs in the closely related model bacteria *P. atrosepticum*, and used RT-qPCR to check their expression stability in contrasting conditions. First, a sequence similarity search was performed between the sequences of reference genes in the genome of *P. atrosepticum*. No significant sequence similarity was found for ABF-0018436 and ABF-0018449. Among the seven remaining genes, five of them presented a high level of sequence similarity in both *P. atrosepticum and D. dadantii*. To determine whether the expression invariability is conserved for these genes in *P. atrosepticum*, we measured the expression of these orthologous genes using real-time RT-qPCR. Four experimental growth conditions were tested in duplicate at both exponential and stationary growth phase in two different growth media (minimal medium supplemented with saccharose and LB rich medium). The results are presented in Figure 6. The five genes were stable in all the growth conditions used. The standard deviation, calculated by Bestkeeper, ranged from 0.05 for *lpxC* (ABL-0064126) to 0.72 for the gene *yhbN* (ABL-0060508). The order of genes (from the most to the least stable) is *lpxC* (ABL-0064126), *yqcD* (ABL-0061261), *hemF* (ABL-0061109), *yafS* (ABL-0063637) and *yhbN* (ABL-0060508). Interestingly, the most stable gene, *lpxC*, in *P. atrosepticum* is the orthologous gene of the most stable gene in *D. dadantii*. Thus, expression of these genes appeared to be stable in the closely related bacteria *P. atrosepticum* and was not subject to growth-phase regulation. They were shown to be as stable as *ffh*, which had been previously demonstrated to be suitable for normalization [18], and so they could be used as alternative reference genes in *P. atrosepticum*. As these genes are largely conserved among Gram-negative bacteria, they could be tested for the stability of their expression in these bacteria.

In conclusion, we have identified reliable reference genes for the normalization of gene expression in *D. dadantii*. Using a microarray transcript profiles dataset, comprising very different growth conditions, we were able to identify expressed genes with very low variation in gene expression. At least two genes, *lpxC* (ABF-0017965) and *yafS* (ABF-0020509), proved to be good candidates as their expression levels are stable in a wide range of growth conditions and during infection of a plant. Normalization of RT-qPCR data of differentially expressed genes with *lpxC* and *yafS* expression levels gave similar expression ratio to those obtained with the microarray data. Moreover, the ortholog of *lpxC* in *P. atrosepticum* also showed stable expression in diverse growth conditions. Thus, these genes are suitable for the normalization of real-time RT-qPCR data, in particular in the two main pectinolytic phytopathogenic bacteria *D. dadantii* and *P. atrosepticum* and, probably, in other related *Enterobacteriaceae*.

Generally, to look for good reference genes from micro-array data, the first criteria is to find genes significantly expressed in a wide range of conditions. Standard deviation of gene expression must be as low as possible. The weak variation in the expression level of the selected genes should not correlate with a specific condition of growth. Finally, to minimize the risk of choosing co-regulated genes, the reference genes must belong to different functional categories and different transcription units. Following these criteria, we assume that good reference genes could be found in any kind of bacteria.

## Methods

### Bacterial strains, cell growth and stress conditions

The *D. dadantii* strain used was 3937 [22] and the *P. atrosepticum* wild-type strain was SCRI1043 [23]. Bacteria were grown in Luria-Bertani (LB) or in minimal salt M63 media, supplemented with various carbon sources (glucose, saccharose, xylose, or saccharose and rhamnose) at 0.2% (w/v). LB and M63 media were prepared as described by [24].

In order to mimic the environment encountered by the bacteria in its host, a multi-factorial design with 32 different experimental conditions was constructed, with two biological replicates for each condition, to study the transcriptome by pangenomic micro-arrays. Cells were grown at exponential *vs* stationary phases in four different growth media: M63 supplemented with 0.2% saccharose as carbon source, with or without 0.2% (w/v) polygalacturonate (PGA) and with or without 0.1% Saintpaulia leaf extracts (1 g leaves in 1 L M63). Cells grown in these four media were subjected to different stresses: (i) acid stress, by an incubation of 15 min in the presence of 30 mM malic acid; (ii) oxidative stress, by an incubation of 15 min in the presence of 100 µM H_2_O_2_ or (iii) osmotic stress, by an incubation of 15 min in the presence of 300 mM NaCl.

Supplementary growth conditions were included in the RT-qPCR experiments: M63 media supplemented with saccharose, or xylose alone at 0.2% (w/v), or both saccharose and rhamnose at 0.2% (w/v) each. When required, media were supplemented with coumermycin to modulate DNA supercoiling: 15 µg/mL were used in LB and 5 µg/mL in M63 medium.

Aerobic cultures were grown, with shaking (150 rpm), at 30°C for *D. dadantii* and *P. atrosepticum* in 500 mL flasks containing 50 mL of medium. A_600_ was determined in a PRIM Secoman spectrophotometer.

### RNA extraction

RNAs from *D. dadantii* were extracted, as previously described [19], from cultures grown to the beginning of exponential phase (A_600_ = 0.2) and to the beginning of stationary phase (A_600_ = 1.5 for cells grown in M63 medium and A_600_ = 1.8 for cells grown in LB medium). RNA samples from *P. atrosepticum* were extracted following the hot phenol protocol previously described by [25]. Purified RNA was treated with DNase I (Roche). Isolated RNA was quantified on the basis of its absorption at 260 nm using an ND 100 Nanodrop spectrophotometer, visualized on an agarose gel to check quality and stored at −80°C until further use. The absence of genomic DNA contamination was checked by PCR with all primer pairs used for quantitative RT-PCR.

### RNA extraction from bacteria infecting plants

Bacteria cells were suspended in a 100 mM KCl solution to an OD_600_ of 0.1, corresponding to a concentration of 10^8^ cfu/ml. Plant leaves were soaked in the bacterial suspension. Plants were incubated in tropical conditions. At 6 hpi (hours post-infection) epiphytic bacteria present on the leaf surface were recovered by rinsing the infected rosettes in an RNA blocking reagent. At this stage, bacteria were isolated from a mean of 130 plants. At 24 hpi, bacteria in a virulent state were recovered by rinsing the fully macerated plant tissues in an RNA blocking reagent. Bacteria were isolated from a mean of 85 plants by differential centrifugation. Total RNA was extracted. Purified RNA was treated with DNase I (Roche). Isolated RNA was quantified on the basis of its absorption at 260 nm using an ND 100 Nanodrop spectrophotometer, visualized on an agarose gel to check quality and stored at −80°C until further use. The absence of plant RNA contamination was checked by PCR with primer pairs specific to the transcription factor EF1 gene and the 23S chloroplastic gene. This was considered to be negligible (data not shown). The absence of genomic DNA contamination was checked by PCR with all primer pairs used for quantitative RT-PCR.

### Micro-arrays experiment and data analysis

The micro-arrays used in this study were custom designed and produced by Roche NimbleGen, Inc. (Madison, WI) based on the annotated sequence of *D. dadantii*, available at Genbank accession number n° CP002038, which comprises 4597 CDS. The 4plex expression micro-arrays consist of 60-mer oligonucleotides, triplicated in three blocks on the array (5 oligonucleotides per CDS). For micro-array analyses, cDNA was synthesized, labeled and hybridized by Roche NimbleGen Inc. Expression data were normalized using quantile normalization [26] and summarized to one expression value per gene, for each array, using the Robust Multichip Average (RMA) algorithm [27].

### Selection of reference gene candidates

We applied the following criteria to select for candidate reference genes. Initially, we chose genes with a mean expression level above an arbitrary threshold (genes likely to be expressed) and with the lowest dispersion of expression across the 64 arrays. Next, we retained those genes (i) associated with different functional categories (to minimize the risk of picking up co-regulated genes); (ii) representing different levels of expression (low, medium and high gene expression); and (iii) showing no systematic variation, however small, correlated with any of the growth condition factors. This approach is very similar to that applied by Andersen *et al.* (2004)

### Reverse transcription, real-time PCR and data analysis

1 µg RNA was retrotranscribed with 50 ng random hexamer using the “RevertAid First strand cDNA synthesis kit” according to the manufacturer's recommendations (Fermentas). In addition, the retrotranscription efficiency was measured by quantification of cDNA produced from the RNA pAW added to 2.10^5^ copies per µL in the reaction of reverse transcription [8], [10], [14]. 0.1 µL of the reverse transcription reaction was added, as template, to the Qbiogen SYBR Green mix for PCR with gene-specific primers. The primers used in this work are listed in Table 2. Primers were tested for their lack of hybridization on the *A. thaliana* genome. The thermal cycling reactions were performed, using the Lightcycler LC 480 from Roche, according to the following conditions: an initial step at 95°C for 10 min, followed by 45 cycles at 94°C for 15 s, 55°C for 20 s and 72°C for 17 s. The specificity of the PCR primers was verified by melting curve analysis.

**Table 2:** The primer set used in this study.

Primers for qPCR expression studies	Forward	Reverse00000
pAW	5′-CATGTCAAATTTCACTGCTTCATC-3′	5′-TGACCACCCAGCCATCCTT-3′
*D. dadantii* ID (gene names)		
14902 (*rpoB*)	5′-CCTTTCAGGTTGAGCAGGAT-3′	5′-AACGTCAAATACCGGCTCAC-3′
18449	5′-CCGAAGGTGAAATGGAGCTA-3′	5′-TTCGGGTCAACATCCAGAAT-3′
20529 (*yafS*)	5′-CTGCGATTGACAGCCAGATA-3′	5′-CCAAACTGACCGGATTGAAC-3′
18436	5′-GACCGACAGCGGTCATTATT-3′	5′-TTGCATCTTTACCGTGGACA-3′
16418 (*ddlA*)	5′-CCTTAAACCGCTCCAGTCAG-3′	5′-CTGCCGATAAACGGAATGTT-3′
15073	5′-ATACGTCAGCACCGAGGAAC-3′	5′-CGATCGTGATAGGCGGTATT-3′
15667 (*yhbN*)	5′-GCCATGAAGTGATCGAAGGT-3′	5′-TCCAGCTGTTCCAGATAGGC-3′
16832 (*yqcD*)	5′-AGCTACCCGGACAAATACGA-3′	5′-TGGACTCGATCAGGTTTTCC-3′
17965 (*lpxC*)	5′-AAATCCGTGCGTGATACCAT-3′	5′-CATCCAGCAGCAGGTAGACA-3′
16748 (*hemF*)	5′-CTGCTCACGTTACAGGACGA-3′	5′-CGGAAACGTGAGAGAAATTCA-3′
20403 (*rraA*)	5′-CGGTGAAATGCTTTGAGGAT-3′	5′-GATGTCCAGTTCCGACAGGT-3′
*P. atrosepticum* ID (gene names)		
61109 (*hemF*)	5′-CTGCTTAGCTTACAGAAAGA-3′	5′-TCACGTGGGAAAAATTTACGC-3′
63637 (*yafS*)	5′-CATGGTGGCCAAAACTCTTT-3′	5′-GATCAACCTCCCGCAGAATA-3′
60508 (*yhbN*)	5′-TTCAGCACAGCAATCTCTGG-3′	5′-AGAACGTCACAGGGTTACCG-3′
61261 (*yqcD*)	5′-TATCACGACCGCTATGATGC-3′	5′-CTGCGTTAAGGTGCATTTCA-3′
64126 (*lpxC*)	5′-ATCTATCGCCGCACAGACTT-3′	5′-GGTGCGTCAACATCAATGAC-3′
63661 (*ffh*)	5′-ATGGGCGATGTGCTTTCACT-3′	5′-TCAAACCCATCGCCTTTCTT-3′

### Stability of gene expression and relative quantification

The stability of gene expression was evaluated using the Excel-based application Bestkeeper [15] and the R package SLqPCR (http://bioconductor.org/packages/release/bioc/html/SLqPCR.html), which reproduced the analysis of the GeNorm method [1].

## Supporting Information

Figure S1 **Expression profiles of the 49 genes selected for their expression stabilities in 32 growth conditions and the expression profiles of** ***ffh*** **,** ***recA*** **,** ***rpoA*** **and** ***rpoB*** **.** Gene expression levels are measured from microarray analyses and are presented as logarithmic values. The first 32 conditions are those measured in exponential phase and the last 32 conditions are those measured in stationary phase. The order of conditions is M63 supplemented with saccharose (S), M63 supplemented with saccharose and Saintpaulia leaves, M63 supplemented with saccharose and PGA, and M63 supplemented with saccharose, leaves and PGA. Stress conditions are presented in black (without any stress), in red for oxidative stress, in green for acid stress and in blue for osmotic stress.
(PDF)

Table S1 GeNorm results as M values.
(DOC)

Table S2 Bestkeeper results.
(DOC)
