# Supplementary material for: lpxC and yafS are the Most Suitable Internal Controls to Normalize Real Time RT-qPCR Expression in the Phytopathogenic Bacteria Dickeya dadantii
Source: PLoS One. 2011 May 26;6(5):e20269. doi: 10.1371/journal.pone.0020269 (PMC3102694; doi:10.1371/journal.pone.0020269)
Supplement: Figure S1 — Expression profiles of the 49 genes selected for their expression stabilities in 32 growth conditions and the expression profiles of ffh , recA , rpoA and rpoB . Gene expression levels are measured from microarray analyses and are presented as logarithmic values. The first 32 conditions are those measured in exponential phase and the last 32 conditions are those measured in stationary phase. The order of conditions is M63 supplemented with saccharose (S), M63 supplemented with saccharose and Saintpaulia leaves, M63 supplemented with saccharose and PGA, and M63 supplemented with saccharose, leaves and PGA. Stress conditions are presented in black (without any stress), in red for oxidative stress, in green for acid stress and in blue for osmotic stress. (PDF) [file pone.0020269.s001.pdf]

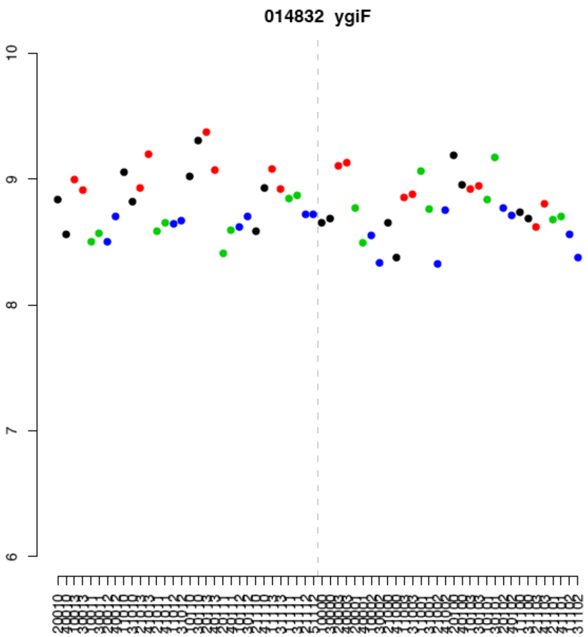
014832 ygiF

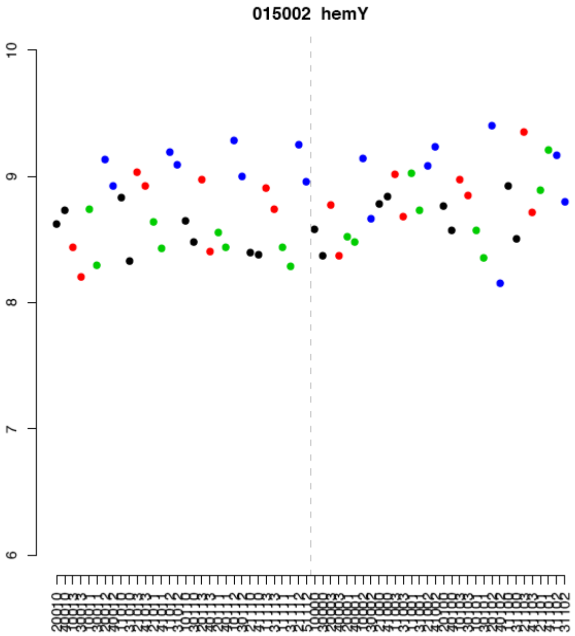
015002 hemY

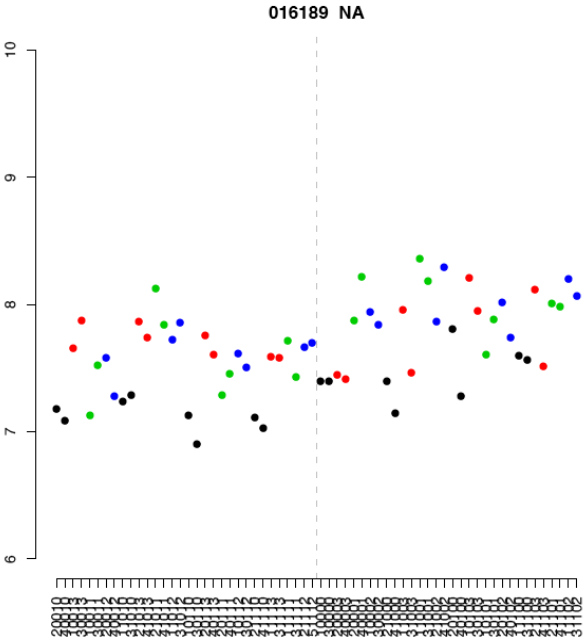
016189 NA

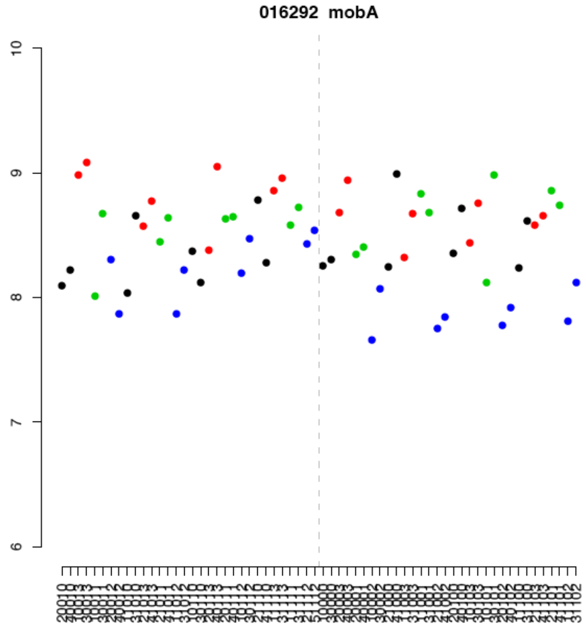
016292 mobA

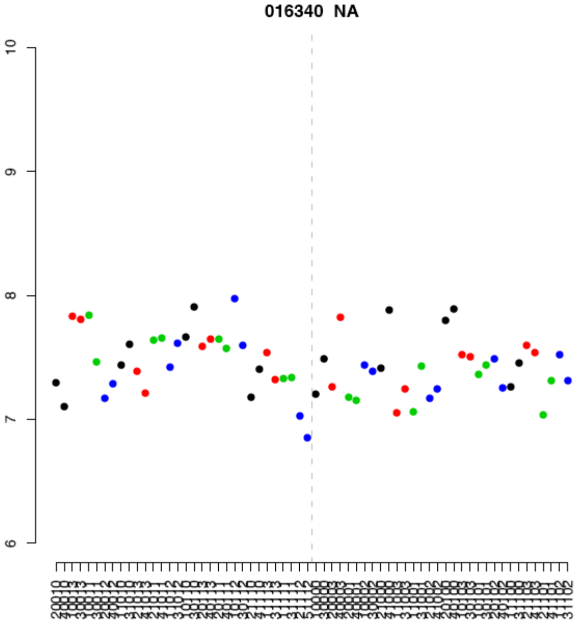
016340 NA

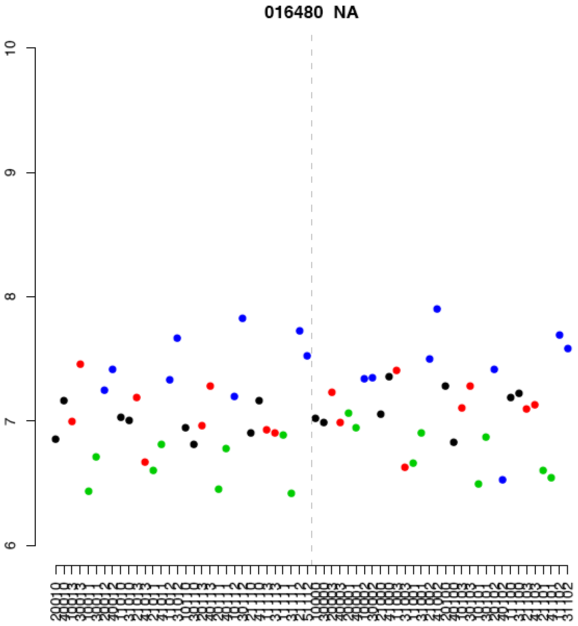
016480 NA

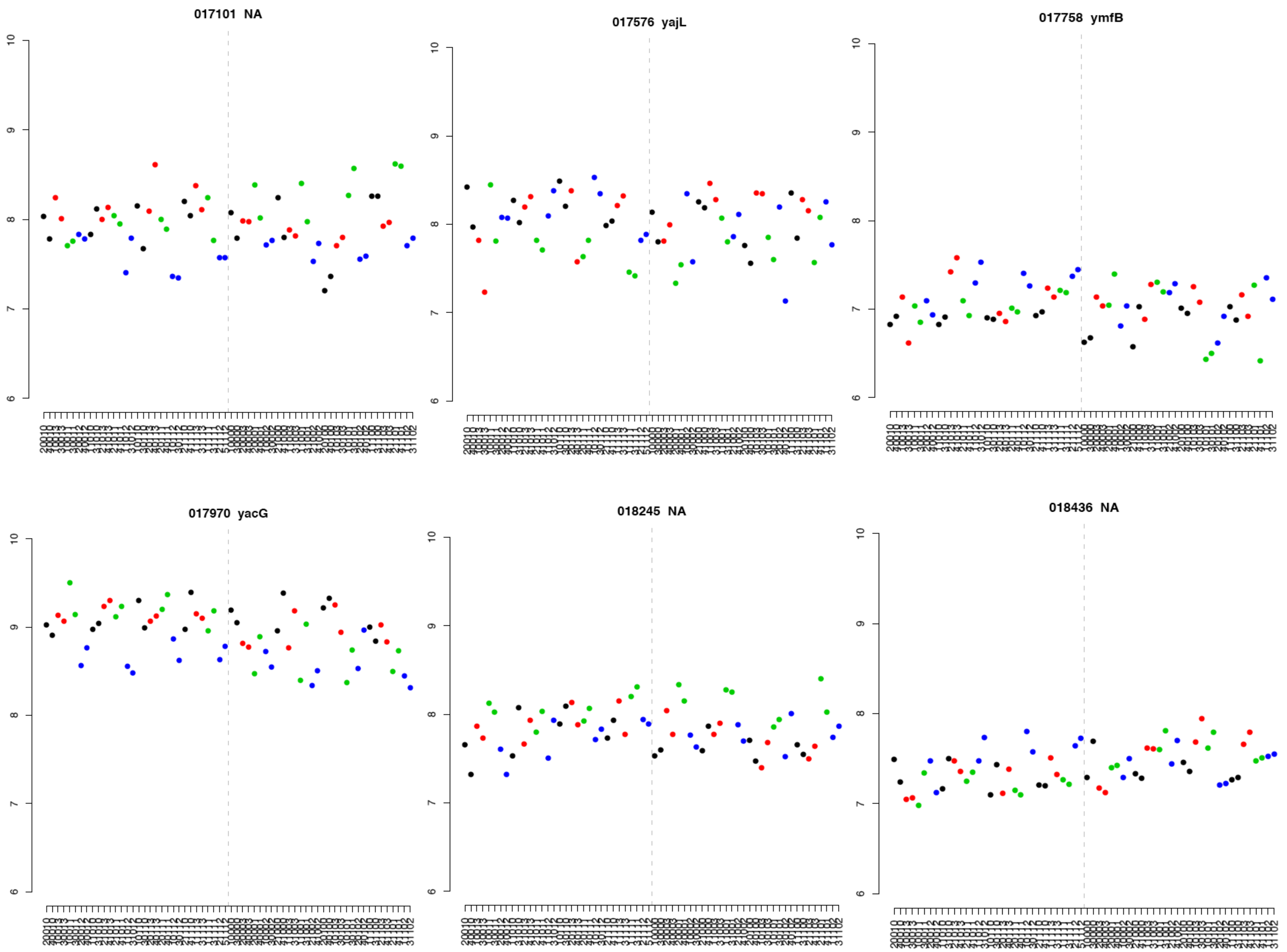
017101 NA
017576 yajL
017758 ymfB
017970 yacG
018245 NA
018436 NA
6
7
8
9
10

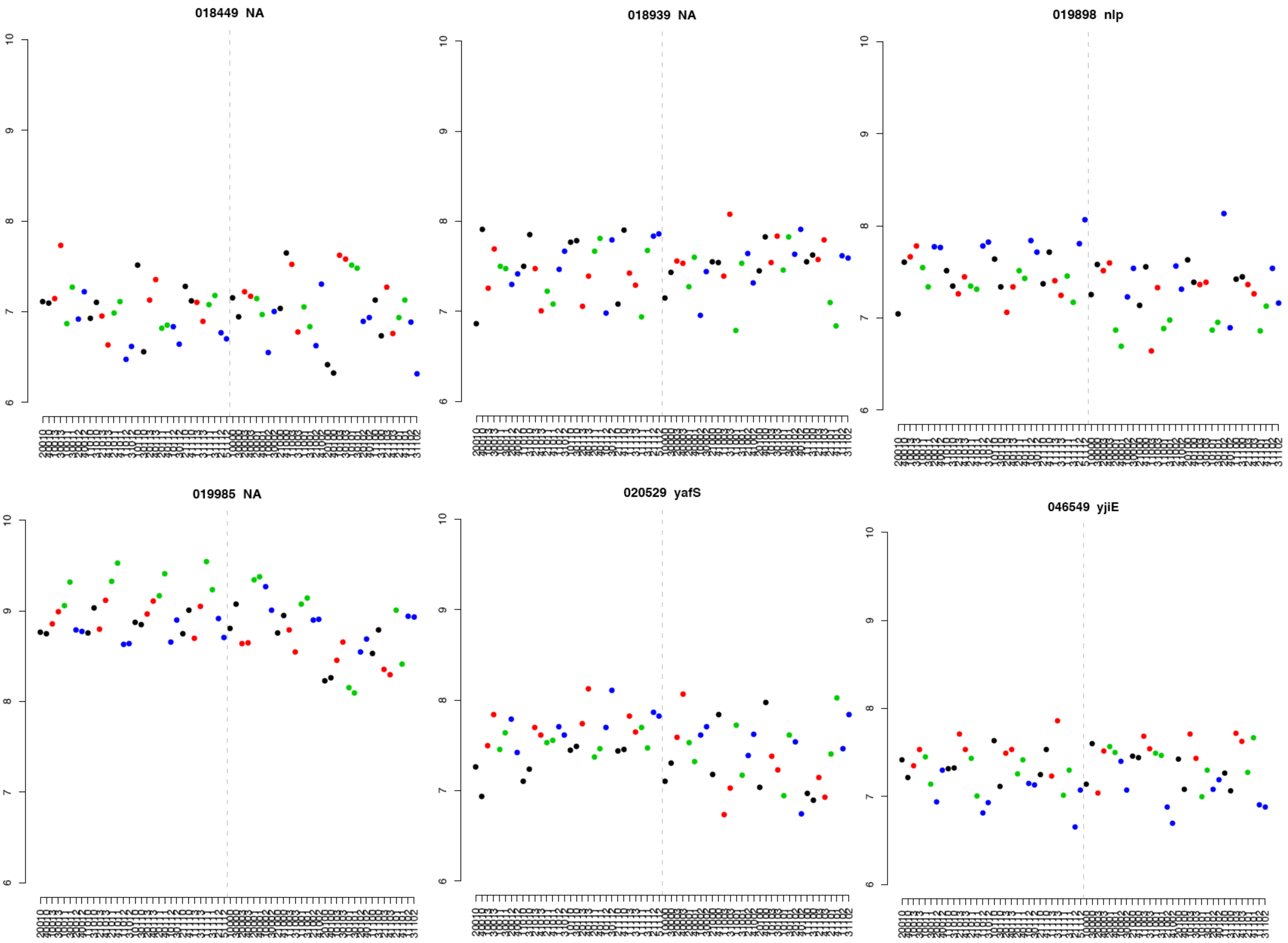
018449 NA
018939 NA
019898 nlp
019985 NA
020529 yafS
046549 yjiE
6
7
8
9
10

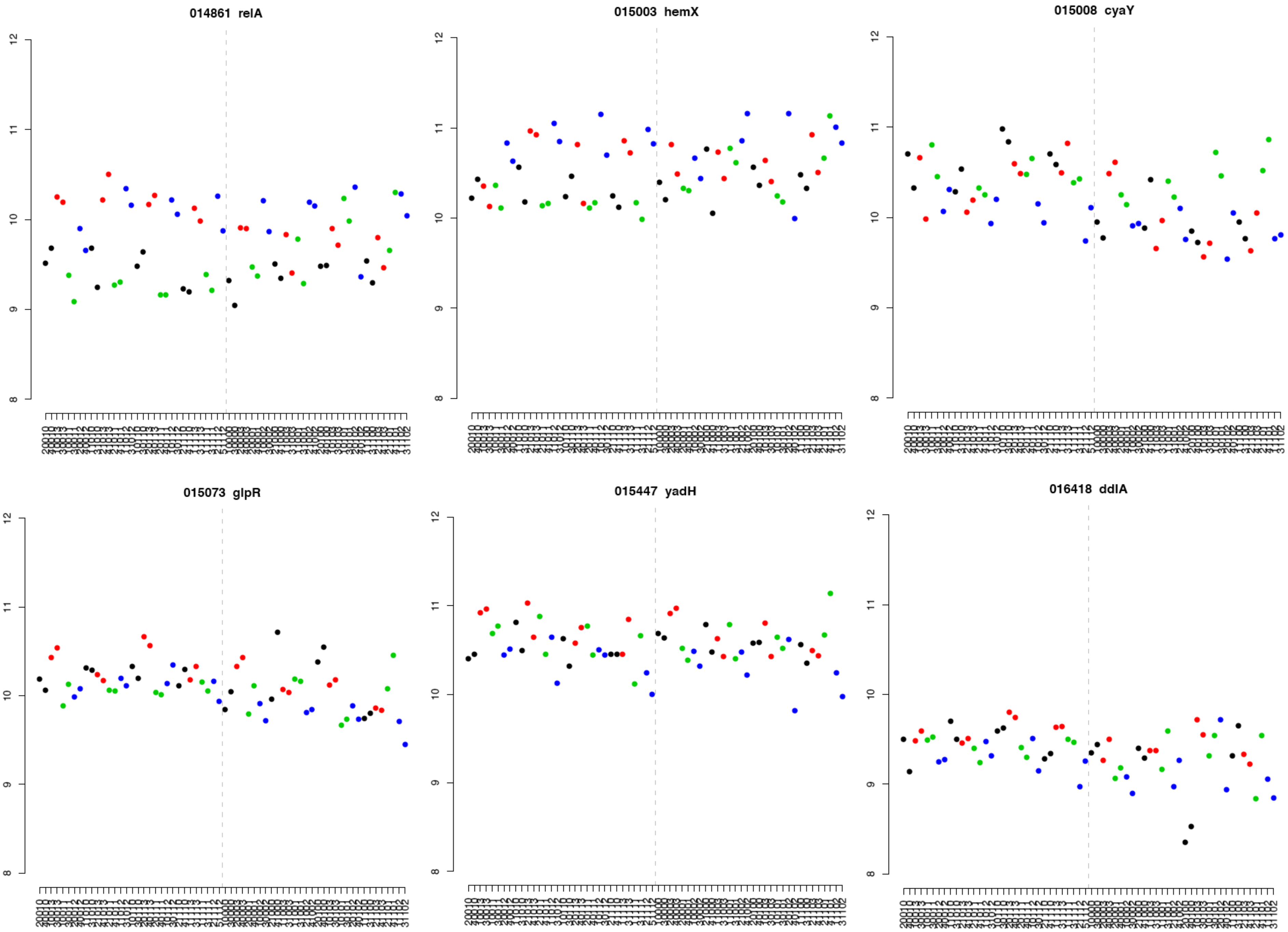
014861 relA
015003 hemX
015008 cyaY
015073 glpR
015447 yadH
016418 ddlA

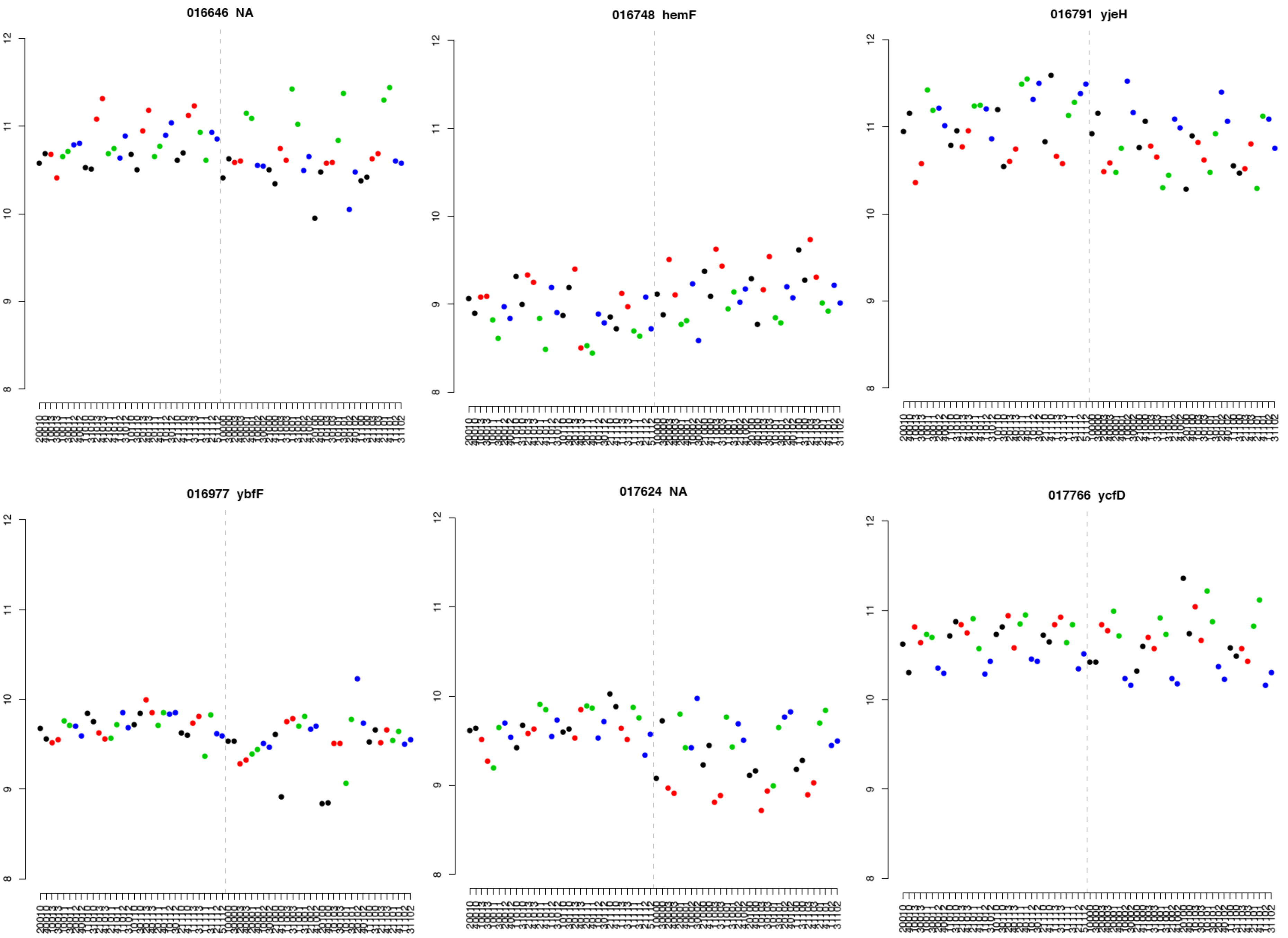
016646 NA
016748 hemF
016791 yjeH
016977 ybfF
017624 NA
017766 ycfD
8
9
10
11
12

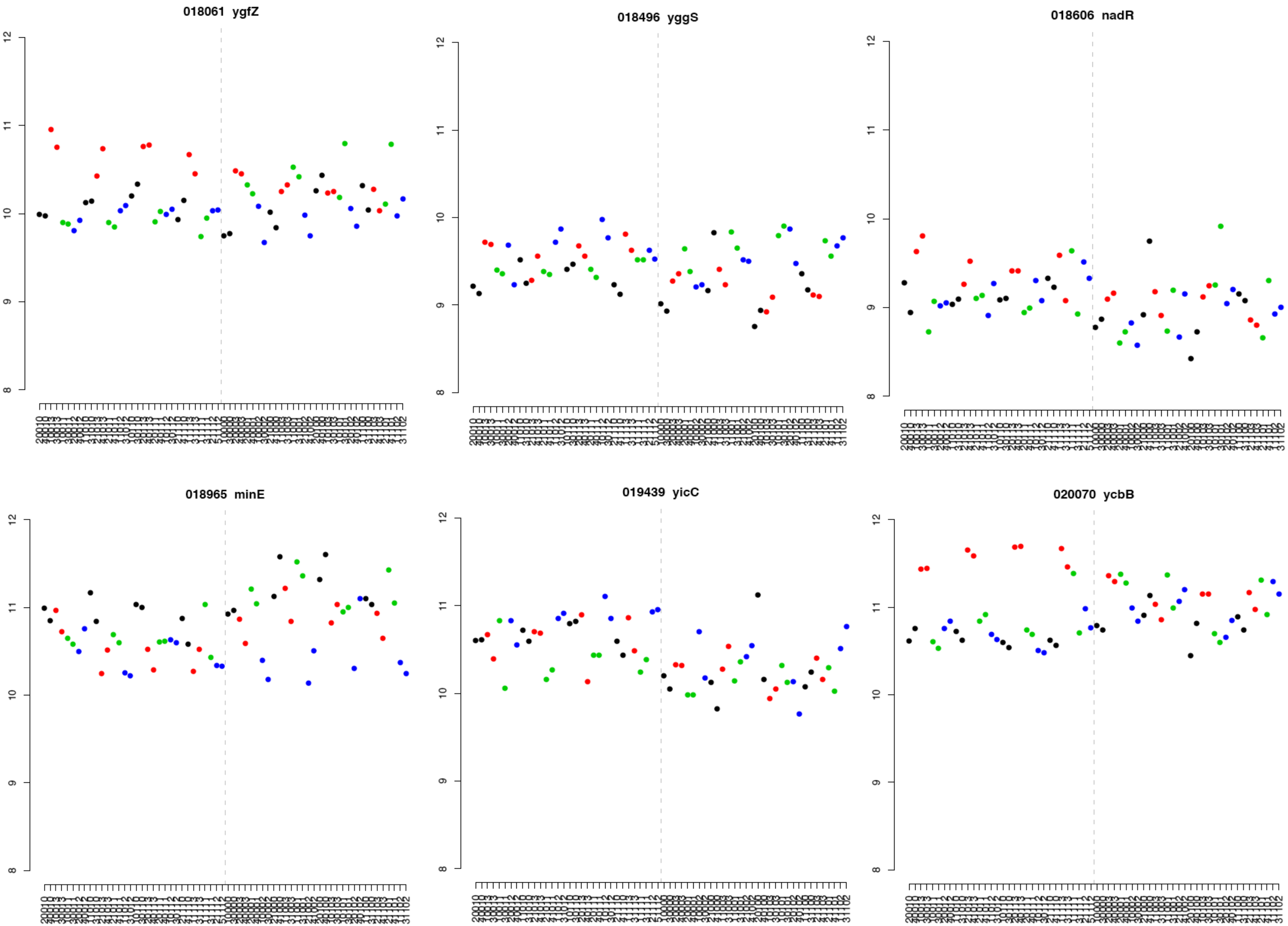
018061 ygfZ
018496 yggS
018606 nadR
018965 minE
019439 yicC
020070 ycbB
8
9
10
11
12

020153 NA

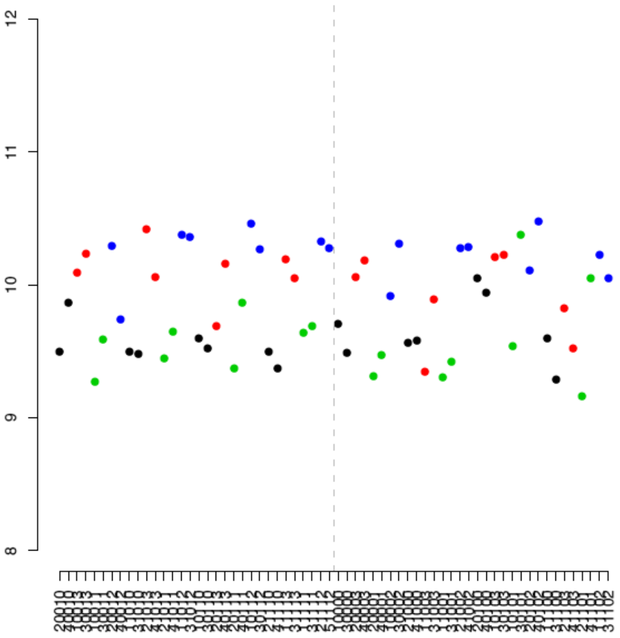

020393 yiiQ

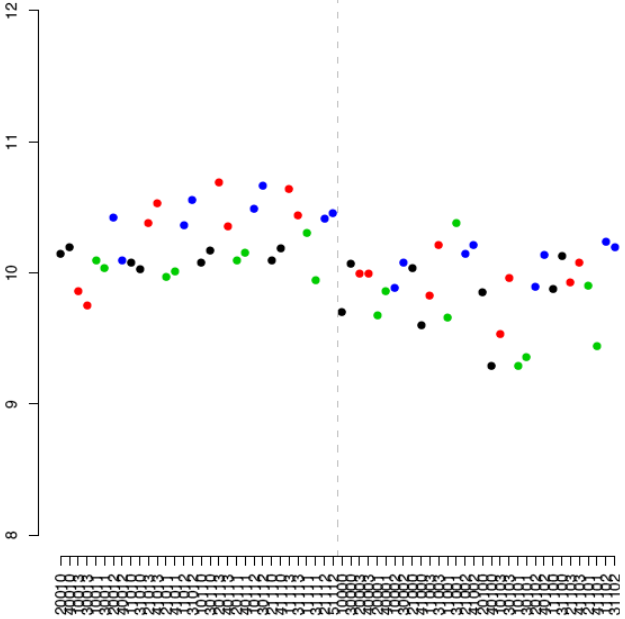

020403 rraA

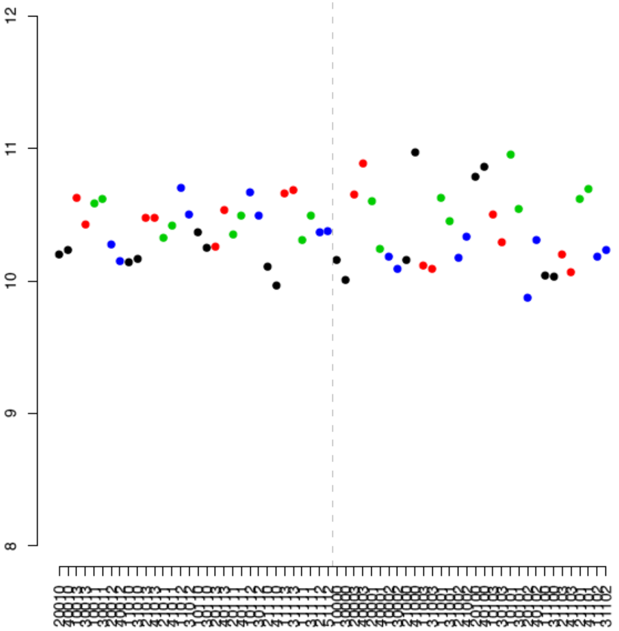

020510 pgsA

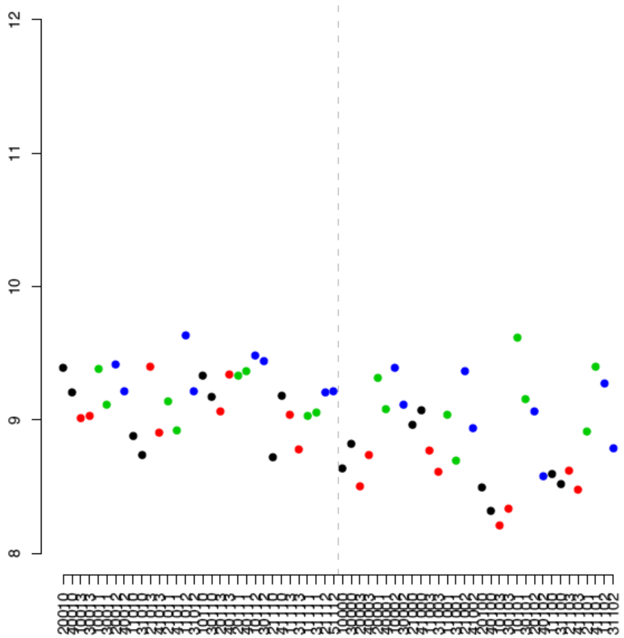

020531 dnaQ

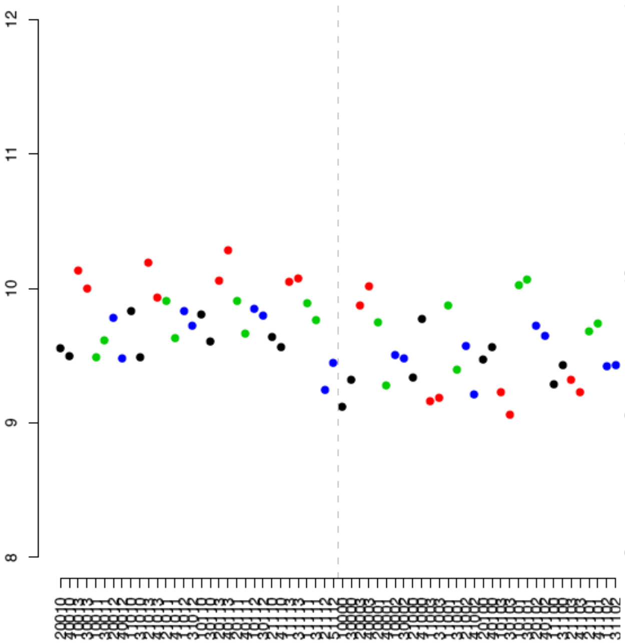

020751 manA

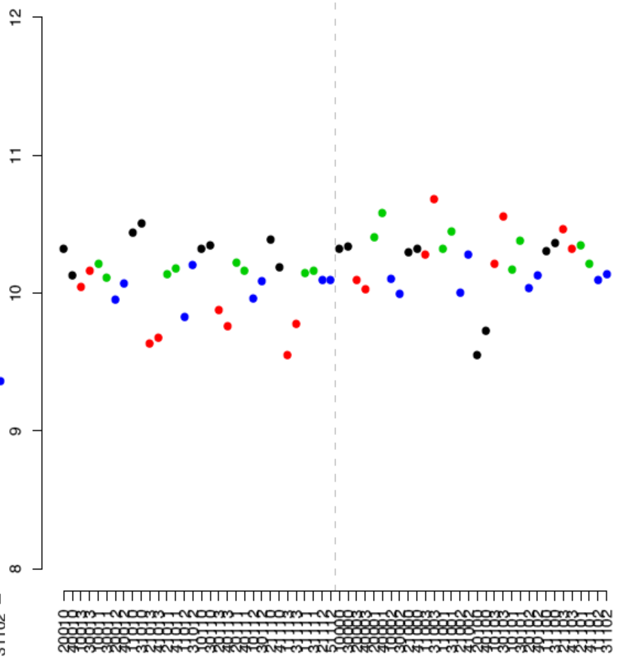

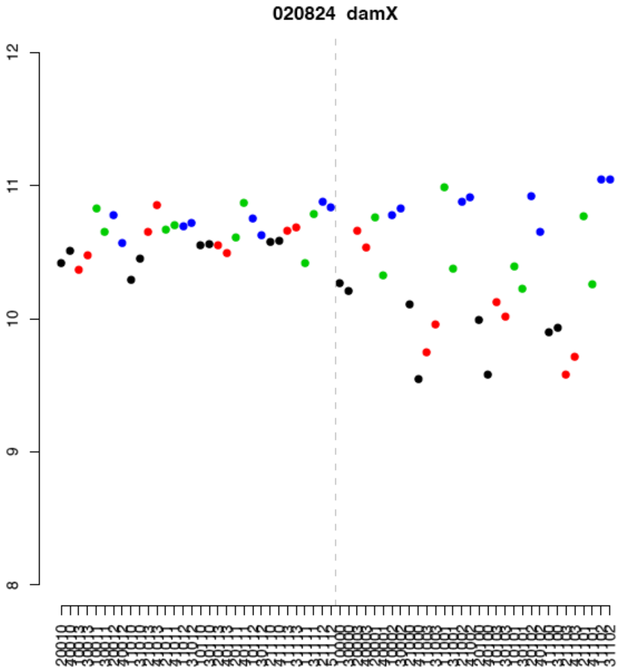
020824 damX
12
11
10
9
8

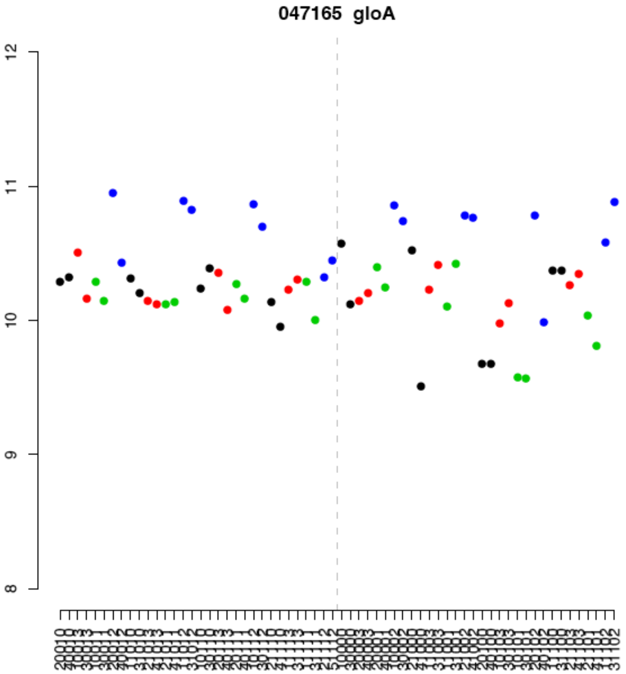
047165 gloA
12
11
10
9
8

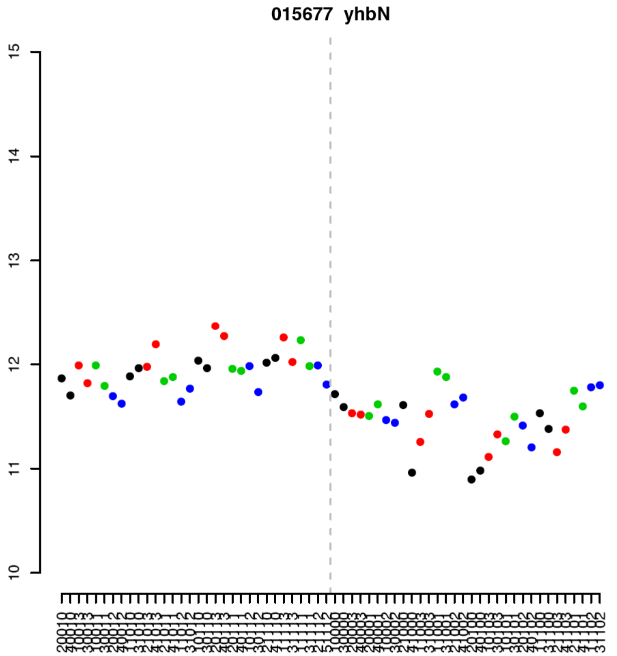
015677 yhbN

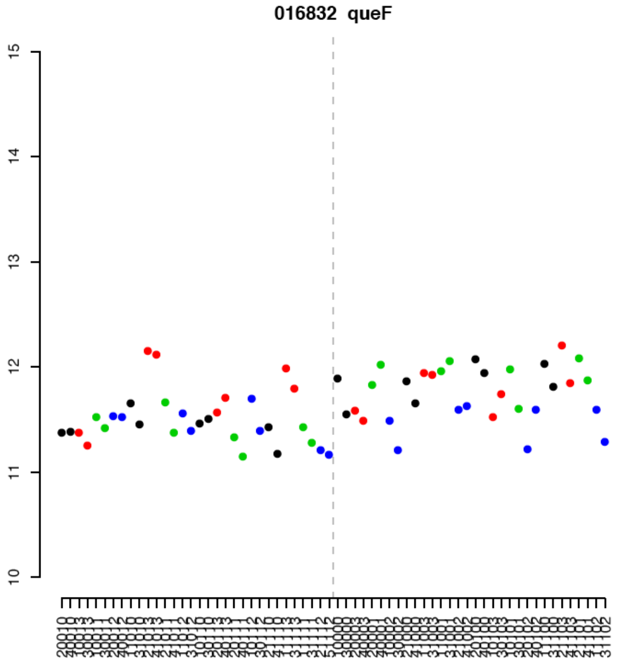
016832 queF

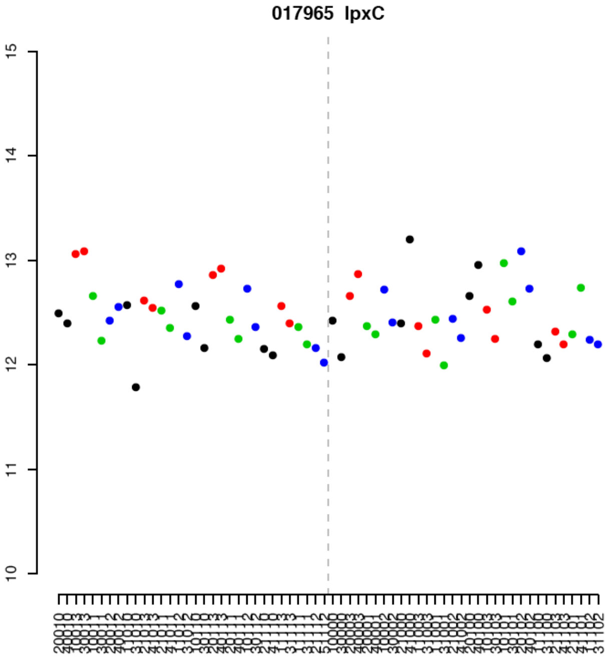
017965 lpxC

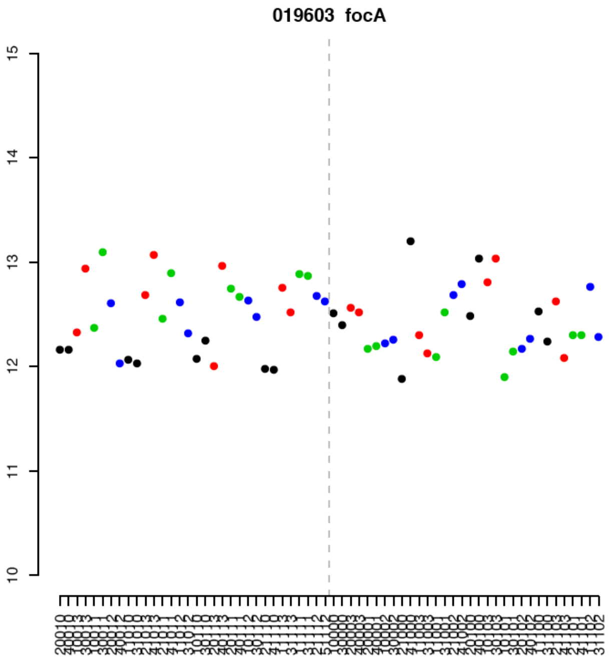
019603 focA

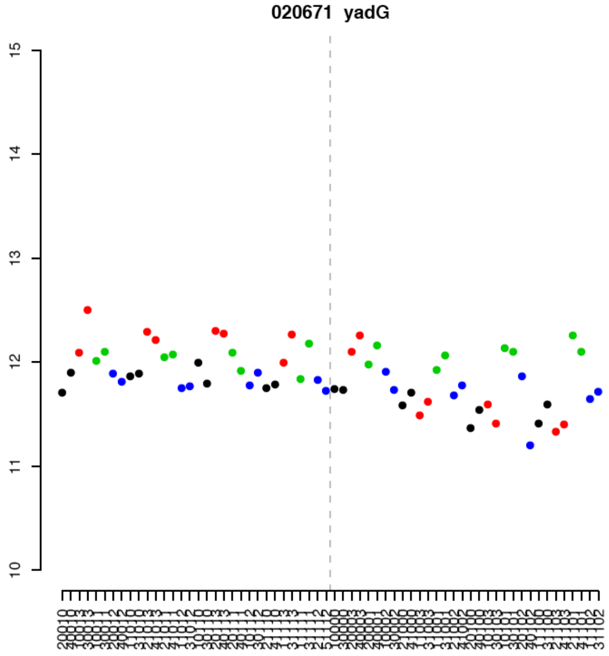
020671 yadG

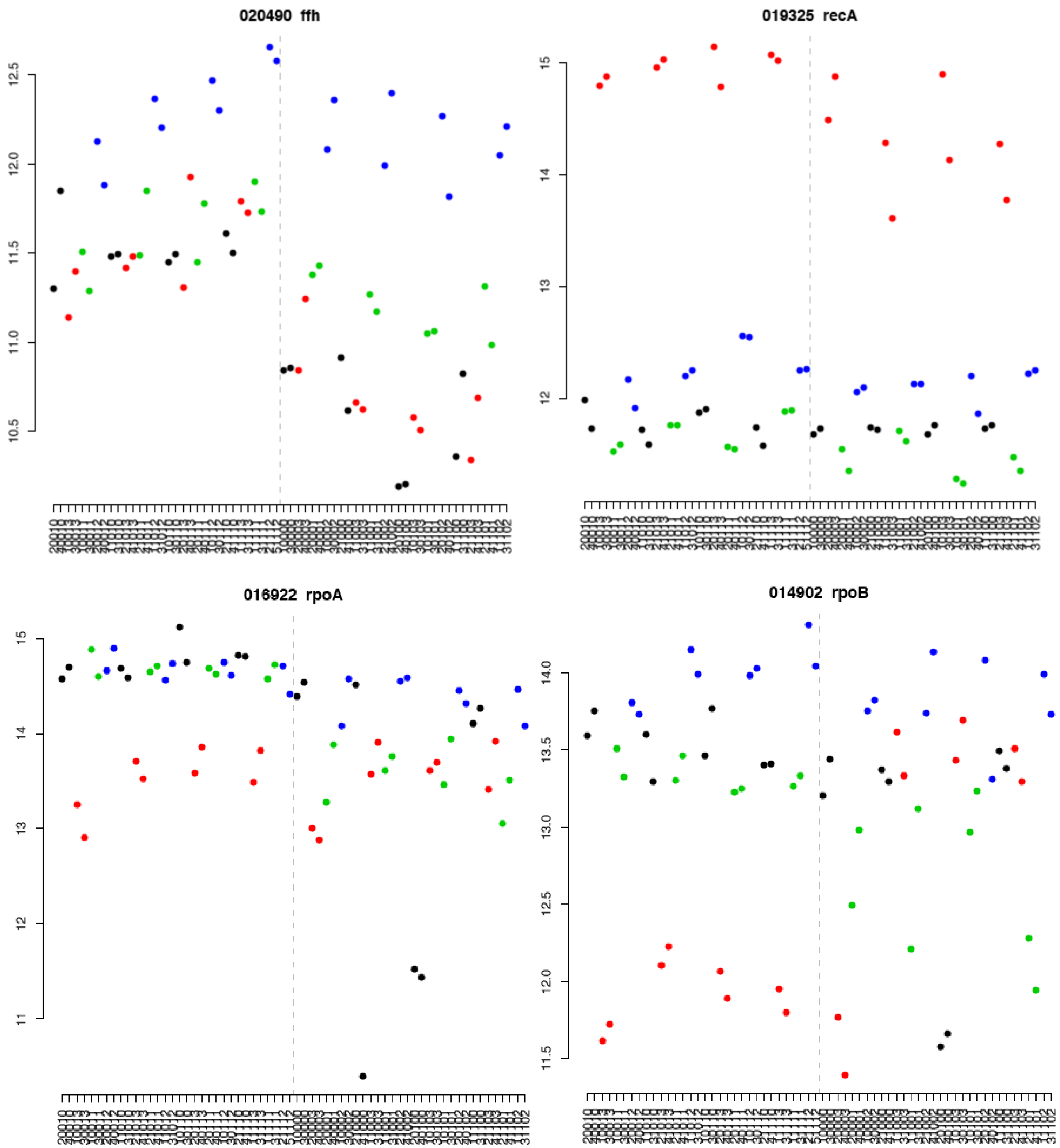
020490 ffh
12.5
12.0
11.5
11.0
10.5
019325 recA
15
14
13
12
016922 rpoA
15
14
13
12
11
014902 rpoB
14.0
13.5
13.0
12.5
12.0
11.5
