# Supplementary material for: lpxC and yafS are the Most Suitable Internal Controls to Normalize Real Time RT-qPCR Expression in the Phytopathogenic Bacteria Dickeya dadantii
Source: PLoS One. 2011 May 26;6(5):e20269. doi: 10.1371/journal.pone.0020269 (PMC3102694; doi:10.1371/journal.pone.0020269)
Supplement: Supplementary file 2 [file pone.0020269.s002.doc]

**A**	ABF-0017965	ABF-0015073	ABF-0020529	ABF-0018436	ABF-0015677	ABF-0018449	ABF-0016832	ABF-0016748	ABF-0016418	ABF-0020403
	0.955	0.997	1.053	1.020	0.930	0.969	0.972	1.030	1.527	2.176
	0.799	0.843	0.897	0.857	0.792	0.847	0.849	0.904	1.399	
	0.713	0.767	0.822	0.789	0.707	0.763	0.767	0.828		
	0.682	0.736	0.780	0.761	0.711	0.788	0.794			
	0.643	0.690	0.732	0.733	0.740	0.858				
	0.593	0.637	0.658	0.675	0.788					
	0.549	0.591	0.605	0.621						
	0.518	0.568	0.599							
	0.487	0.487								

**B**	ABF-0016832	ABF-0017965	ABF-0020403	ABF-0016748	ABF-0015073	ABF-0020529	ABF-0016418	ABF-0018449	ABF-0015677	ABF-0018436
	0.636	0.616	0.682	0.662	0.631	0.651	0.737	0.791	0.854	0.905
	0.630	0.587	0.660	0.665	0.592	0.622	0.678	0.793	0.798	
	0.579	0.559	0.611	0.644	0.611	0.594	0.716	0.747		
	0.570	0.523	0.594	0.613	0.580	0.615	0.668			
	0.537	0.513	0.556	0.592	0.594	0.599				
	0.524	0.484	0.563	0.559	0.610					
	0.461	0.489	0.523	0.556						
	0.439	0.463	0.472							
	0.430	0.430								

**C**	ABF-0018436	ABF-0017965	ABF-0020529	ABF-0016418	ABF-0016748	ABF-0015073	ABF-0020403	ABF-0018449	ABF-0015677	ABF-0016832
	0.810	0.853	0.901	0.871	0.886	0.878	1.097	1.287	1.803	1.841
	0.674	0.709	0.775	0.788	0.798	0.778	0.954	1.319	1.692	
	0.563	0.594	0.623	0.642	0.648	0.697	0.874	1.193		
	0.455	0.468	0.519	0.586	0.617	0.625	0.753			
	0.417	0.465	0.487	0.517	0.562	0.573				
	0.418	0.434	0.441	0.501	0.547					
	0.373	0.374	0.421	0.497						
	0.320	0.290	0.396							
	0.214	0.214								
