# Supplementary material for: lpxC and yafS are the Most Suitable Internal Controls to Normalize Real Time RT-qPCR Expression in the Phytopathogenic Bacteria Dickeya dadantii
Source: PLoS One. 2011 May 26;6(5):e20269. doi: 10.1371/journal.pone.0020269 (PMC3102694; doi:10.1371/journal.pone.0020269)
Supplement: Supplementary file 3 [file pone.0020269.s003.doc]

**A**	ABF-0020529	ABF-0018436	ABF-0018449	ABF-0016832	ABF-0017965	ABF-0016418	ABF-0015073	ABF-0015677	ABF-0016748	ABF-0020403
n	64	64	64	64	64	64	64	64	64	64
geo Mean [Cq]	25.42	30.56	30.09	23.07	18.10	21.60	22.69	21.46	24.08	23.28
ar Mean [Cq]	25.43	30.57	30.11	23.09	18.10	21.66	22.70	21.47	24.10	23.29
min [Cq]	23.29	28.68	28.68	21.26	16.99	19.36	31.35	20.03	22.65	21.74
max [Cq]	27.34	32.75	32.60	25.78	19.47	25.98	24.29	24.45	26.10	25.20
std dev [± Cq]	0.56	0.59	0.68	0.72	0.41	1.22	0.45	0.64	0.72	0.52

**B**	ABF-0020529	ABF-0018436	ABF-0018449	ABF-0016832	ABF-0017965		ABF-0015073	ABF-0015677	ABF-0016748	ABF-0020403
n	10	10	10	10	10		10	10	10	10
geo Mean [Cq]	27.55	33.20	30.68	23.38	19.88		24.14	22.39	23.92	24.84
ar Mean [Cq]	27.59	33.26	30.71	23.49	19.89		24.28	22.49	23.94	24.87
min [Cq]	26.04	31.53	29.30	22.15	19.06		23.00	20.49	23.07	23.62
max [Cq]	31.97	39.20	33.88	30.65	21.68		28.41	28.86	27.30	28.42
std dev [± Cq]	0.94	1.29	0.85	1.43	0.45		0.95	1.44	0.69	0.84

**C**	ABF-0020529	ABF-0018436	ABF-0018449	ABF-0016832	ABF-0017965		ABF-0015073	ABF-0015677	ABF-0016748	ABF-0020403
n	6	6	6	6	6		6	6	6	6
geo Mean [Cq]	26.03	30.60	27.24	19.60	18.55		22.20	36.74	27.42	28.19
ar Mean [Cq]	26.04	30.60	27.26	19.66	18.56		22.20	36.76	27.42	28.20
min [Cq]	25.04	29.95	26.19	17.91	17.59		21.61	35.53	26.89	27.11
max [Cq]	27.02	31.10	28.71	22.83	19.15		22.86	39.50	27.98	28.79
std dev [± Cq]	0.5	0.36	0.73	1.07	0.45		0.40	1.07	0.36	0.62
